# Association Study of Polymorphisms rs4552569 and rs17095830 and the Risk of Ankylosing Spondylitis in a Taiwanese Population

**DOI:** 10.1371/journal.pone.0052801

**Published:** 2013-01-04

**Authors:** James Cheng-Chung Wei, Yu-Wen Hsu, Kuo-Sheng Hung, Ruey-Hong Wong, Chun-Huang Huang, Yi-Tzu Liu, Yuh-Cherng Guo, Shiro Ikegawa, Wei-Chiao Chang

**Affiliations:** 1 Division of Allergy, Immunology and Rheumatology, Department of Medicine, Chung Shan Medical University Hospital, Taichung, Taiwan; 2 Institute of Medicine, Chung Shan Medical University, Taichung, Taiwan; 3 Department of Clinical Pharmacy, School of Pharmacy, Taipei Medical University, Taipei, Taiwan; 4 Department of Neurosurgery, Center of Excellence for Clinical Trial and Research, Graduate Institute of Injury Prevention and Control, Taipei Medical University, Wan Fang Medical Center, Taipei, Taiwan; 5 Department of Public Health, Chung Shan Medical University, Taichung, Taiwan; 6 Department of Neurology, Changhua Christian Hospital, Changhua, Taiwan; 7 Laboratory for Bone and Joint Diseases, Center for Genomic Medicine, RIKEN, Tokyo, Japan; 8 Department of Pharmacy, Taipei Medical University-Wanfang Hospital, Taipei, Taiwan; Oregon Health & Science University, United States of America

## Abstract

Ankylosing spondylitis (AS) is a chronic inflammation of the sacroiliac joints, spine and peripheral joints. However, the development of anklosing spondylitis is unclear. Human leukocyte antigens HLA-B27 and ERAP1 have been widely reported to be associated with AS susceptibility. A recent genome-wide association study (GWAS) showed that two new susceptibility loci between *EDIL3* and *HAPLN1* at 5q14.3 (rs4552569) and within *ANO6* at 12q12 (rs17095830) contribute to the risk of AS in Han Chinese. In this study, we enrolled 475 AS patients and 475 healthy subjects to assess whether these genetic variations contribute to the susceptibility and the severity of AS in the Taiwanese population. The correlation between genetic polymorphisms, AS activity indexes, (namely, BASDAI, BASFI and BAS-G) and AS complications (uveitis and inflammatory bowel disease) were tested using the markers, rs4552569 and rs17095830. Although no association between rs4552569/rs17095830 genetic polymorphisms and AS susceptibility/severity was found, a significant association between rs17095830 and inflammatory bowel disease was observed in a Taiwanese population.

## Introduction

Ankylosing spondylitis (AS), a chronic inflammation disorder, affects the sacroiliac joints, lumbar spine, and peripheral joints [1]. It occurs predominately in men than in women. [2] The development of AS has not been completely clarified. Many mechanisms are still ambiguous and less understood. AS is strongly associated with the human leukocyte antigen *HLA-B27* gene [3], but *HLA-B27* accounts for only 16% of the genetic variability in AS [4]. Compared with healthy subjects, AS patients show higher expression of circulating CD4^+^ T cells and CD8^+^ T cells [5]–[6]; therefore, the abnormal expression of these T cells may also be associated with AS.

It has been proven that *HLA-B60*, *B61* and *IL-1*, and *IL-23R* play an important role in AS pathogenesis [7], [8], [9]. The combined genotypes of *PD-1* G-536A, *PD-L1* A8923C and *PD-L2* C47103T show an association with AS development [10]. To elucidate the complexity of AS, the identification of genetic factors related to AS susceptibility may be useful. A recent genome-wide association study (GWAS) indicated that two new susceptibility loci between *EDIL3* and *HAPLN1* at 5q14.3 (rs4552569) and within *ANO6* at 12q12 (rs17095830) contribute to the risk of AS in Han Chinese [11].

In this study, we assessed whether these genetic variations contribute to the susceptibility of AS in the Taiwanese population. We further investigated the genetic association with AS complications (uveitis and inflammatory bowel disease) and AS activity (BASDAI, BASFI, and BAS-G) by using the markers of rs4552569 and rs17095830. Our results imply that rs4552569 and rs17095830 may not associate with the susceptibility of AS. However, a significant association between *ANO6* polymorphism rs17095830 and inflammatory bowel disease (IBD) was observed.

## Materials and Methods

### Patients studied

AS patients who fulfilled the selection criteria, were solicited sequentially at Chung Shan Medical University Hospital in Taichung, Taiwan. Before any data, we collected the informed consent from the respondents. AS patients were recruited by three selection criteria: (a) patients aged 16–65 years; (b) cognitive performance not influenced by other diseases such as dementia; and (c) AS diagnosis by the modified New York criteria [12]. AS diagnosed by a qualified rheumatologist and sacroilitis was confirmed by a qualified radiologist. The detailed clinical history included extraspinal manifestations, age on initial symptom, and family history of AS. When the first symptom (axial symptom, peripheral arthritis, uveitis or enthesitis) had developed, age of AS symptom onset was defined as the time. Peripheral arthritis was defined as the presence of at least one swollen joint. The presence of the inflammatory condition of the colon and small intestine, including ulcerative colitis and Crohn's disease was defined as inflammatory bowel disease (IBD) (distinct from irritable bowel syndrome). Uveitis was defined as the inflammation of the middle layer of the eye, which involved patterns as unilateral, bilateral, or alternative. These symptoms were recorded in medical record reviews, and were ascertained by the rheumatologist, ophthalmologist and gastroenterologist. All of AS patients in this study have sacroiliitis. The study was approved by the Institute Review Board of Chung Shan Medical University Hospital, and the design of the work and final report conformed to the Declaration of Helsinki. All the subjects gave the written consent form.

### Bath Ankylosing Spondylitis Indices

Bath Ankylosing Spondylitis Disease Activity Index (BASDAI), Bath Ankylosing Spondylitis Functional Index (BASFI), and Bath Ankylosing Spondylitis Global (BAS-G) were applied to evaluate the disease activity, physical function and global wellbeing, respectively. The modified Chinese versions of BASDAI, BASFI, and BAS-G have good intra-class correlation and Cronbach's alpha [13].

### Genotyping

Genotyping for two SNPs was carried out using the TaqMan Allelic Discrimination Assay (Applied Biosystems, Foster city, CA) as our previous report [14]. The polymerase chain reaction (PCR) was using a 96-well microplate with the ABI9700 Thermal Cycler. After PCR, the System SDS software version 1.2.3 was used to detected and analyzed fluorescence.

### Statistical analysis

JMP 8.0 for Windows was used for analysis. Hardy-Weinberg equilibrium was assessed by the χ^2^ test with 1 degree of freedom. The statistical differences between the patient and control groups, in genotype and allele frequency were analyzed by the chi-square test. Among different genotypes in AS patients, analysis of variance (ANOVA) was used to compare the mean of continuous variables (BASDAI, BASFI, and BAS-G). Multiple regression analysis was used to adjust for age, sex and disease duration. The Bonferroni test was used to correct for multiple tests. It is considered as significant when a *P* value is less than 0.025.

## Results

### No association of rs4552569 and rs17095830 genetic polymorphisms in AS susceptibility

A total of 475 patients with AS and 475 healthy subjects were recruited. All AS patients were diagnosed according to the modified New York criteria (mean age, 39 years; men %, 68). In our AS cohort, 90.7% were HLA-B27 positive. Potential controls were randomly selected from sequential patients with no significant medical histories or abnormal laboratory results. All individuals were Taiwanese and gave informed written consent to participate in the study. As shown in the Table 1, no significant association between the genetic variants (rs4552569/rs17095830) and AS was observed. We also considered 12 combinatorial patterns of these two SNPs (as shown in Figure S1) and performed association studies of the susceptibility in AS patients, however, none of significant association was observed (data not shown).

**Table 1:** Genotype and allele frequencies of rs4552569 and rs17095830 in controls and patients with AS.

	Genotype	Case (%)(n = 475)	Control (%)(n = 475)	Allele	Case (%)(n = 475)	Control (%)(n = 475)	Genotype*P* Value	Dominant*P* Value	Recessive*P* Value	Allelic*P* Value
rs4552569	CC	32 (6.8)	40 (9.1)	C	258 (27.4)	245 (27.9)	0.3033	0.6754	0.1988	0.8275
	CT	194 (41.3)	165 (37.6)	T	682 (72.6)	633 (72.1)				
	TT	244 (51.9)	234 (53.3)							
rs17095830	GG	6 (1.3)	5 (1.1)	G	96 (10.2)	77 (8.7)	0.5266	0.2583	0.8404	0.2731
	AG	84 (17.8)	67 (15.1)	A	848 (89.8)	811 (91.3)				
	AA	382 (80.9)	372 (83.8)							

### rs17095830 associated with the inflammatory bowel disease in AS patients

We further investigated whether these two genetic polymorphisms associated with the complications (uveitis and IBD) and clinical phenotypes including Bath Ankylosing Spondylitis Disease Activity Index (BASDAI), Bath Ankylosing Spondylitis Functional Index (BASFI), and Bath Ankylosing Spondylitis Global Index (BAS-G). As shown in Table 2, we failed to find the association between two SNPs and uveitis. However, a significant association between *ANO6* polymorphism rs17095830 and inflammatory bowel disease (IBD) was found even after Bonferroni correction (Table 3). Importantly, if the combinatorial effects by two locus model (rs4552569 and rs17095830) were considered, a borderline association (P = 0.0266) was obtained in BASDAI (Table 4).

**Table 2:** Genotype and allele frequencies of rs4552569 and rs17095830 in AS patients with or without uveitis.

	Genotype	Uveitis (%)(n = 46)	Without (%)(n = 360)	Allele	Uveitis (%)(n = 46)	Without (%)(n = 360)	Genotype*P* Value	Dominant*P* Value	Recessive*P* Value	Allelic*P* Value
rs4552569	CC	3 (6.7)	25 (7.0)	C	30 (33.3)	192 (26.9)	0.2085	0.0944	0.9335	0.1976
	CT	24 (53.3)	142 (39.8)	T	60 (66.7)	522 (73.1)				
	TT	18 (40.0)	190 (53.2)							
rs17095830	GG	0 (0.0)	5 (1.4)	G	7 (7.8)	73 (10.2)	0.6762	0.5764	0.4250	0.4697
	AG	7 (15.6)	63 (17.6)	A	83 (92.2)	643 (89.8)				
	AA	38 (84.4)	290 (81.0)							

**Table 3:** Genotype and allele frequencies of rs4552569 and rs17095830 in AS patients with or without inflammatory bowel disease (IBD).

	Genotype	IBD (%)(n = 23)	Without (%)(n = 400)	Allele	IBD (%)(n = 23)	Without (%)(n = 400)	Genotype*P* Value	Dominant*P* Value	Recessive*P* Value	Allelic*P* Value
rs4552569	CC	3 (13.0)	26 (6.6)	C	14 (30.4)	218 (27.5)	0.4524	0.9510	0.2341	0.6681
	CT	8 (34.8)	166 (41.9)	T	32 (69.6)	574 (72.5)				
	TT	12 (52.2)	204 (51.5)							
rs17095830	GG	0 (0.0)	6 (1.5)	G	9 (19.6)	78 (9.8)	**0.0213** *	**0.0131** *	0.5526	0.0350
	AG	9 (39.1)	66 (16.6)	A	37 (80.3)	716 (90.2)				
	AA	14 (60.9)	325 (81.9)							

* Significant (P<*0.025*) values are in bold.

**Table 4:** Difference in the scores of BASDAI, BASFI, and BAS-G among AS patients stratified by rs4552569 and rs17095830 genotypes.

SNP	Genotype	BASDAI	BASFI	BAS-G
rs4552569	CC	4.89±1.92	2.24±2.05	4.38±2.46
	CT	4.34±2.22	2.07±2.22	4.52±2.79
	TT	4.20±2.20	2.01±2.18	4.27±2.75
Unadjusted *P*-value		0.2393	0.8297	0.6436
Adjusted *P*-value		0.2387†	0.7625§	0.6405†
rs17095830	GG	3.91±3.23	2.93±4.39	4.12±4.62
	AG	4.62±2.22	2.09±2.08	4.58±2.94
	AA	4.26±2.18	2.04±2.19	4.36±2.68
Unadjusted *P*-value		0.3610	0.6202	0.7779
Adjusted *P*-value		0.3602†	0.9494§	0.7757†
**Two-locus model**				
High-risk group		4.34±2.19^b^	2.17±2.28^b^	4.49±2.94^b^
Low-risk group		4.01±2.17^b^	2.02±2.17^b^	4.36±2.71^b^
Unadjusted *P*-value		**0.0266**	0.5832	0.7235
Adjusted *P*-value		**0.0264** †	0.5077§	0.7222†

Data represent means ± S.D..

† Adjusted the effects of age and sex.

§ Adjusted the effects of age, sex and disease duration. 0.025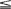 *P*<0.05 values are in bold.

b AS patients with the CC genotype of rs4552569, GG genotype of rs17095830 or heterozygous at both loci were classified into high-risk group and the others were classified into low- risk group.

### No association of rs4552569 and rs17095830 genetic polymorphisms in controls and patients among HLA-B27 (+) with AS

We performed analysis between HLA-B27 (+) AS patients and controls with genetic variants rs4552569 and rs17095830, but we did not observe a significant association (Table 5). In HLA-B27 (+) AS patients, we examined the complications of AS and the association with rs4552569 and rs17095830, however our result showed no statistic significance after Bonferroni correction (Table 6 and Table 7). Our results also revealed no association in the BASDAI, BASFI, and BAS-G scores among HLA-B27 (+) AS patients with the rs4552569 and rs17095830 genotypes (Table 8). Analysis by two locus model didn't improve the significance (Table 8). Thus, our results suggest that rs4552569 and rs17095830 may not associate with AS in a Taiwanese population.

**Table 5:** Genotype and allele frequencies of rs4552569 and rs17095830 in controls and patients among HLA-B27 (+) with AS.

	Genotype	Case (%)(n = 431)	Control (%)(n = 475)	Allele	Case (%)(n = 431)	Control (%)(n = 475)	Genotype*P* Value	Dominant*P* Value	Recessive*P* Value	Allelic*P* Value
rs4552569	CC	27 (6.3)	40 (9.1)	C	231 (27.1)	245 (27.9)	0.2160	0.7259	0.1271	0.7124
	CT	177 (41.6)	165 (37.6)	T	621 (72.9)	633 (72.1)				
	TT	222 (52.1)	234 (53.3)							
rs17095830	GG	5 (1.2)	5 (1.1)	G	88 (10.3)	77 (8.7)	0.4588	0.2200	0.9535	0.2510
	AG	78 (18.2)	67 (15.1)	A	768 (89.7)	811 (91.3)				
	AA	345 (80.6)	372 (83.8)							

**Table 6:** Genotype and allele frequencies of rs4552569 and rs17095830 in HLA-B27 (+) AS patients with or without uveitis.

	Genotype	Uveitis (%)(n = 39)	Without (%)(n = 330)	Allele	Uveitis (%)(n = 39)	Without (%)(n = 330)	Genotype*P* Value	Dominant*P* Value	Recessive*P* Value	Allelic*P* Value
rs4552569	CC	2 (5.3)	21 (6.4)	C	23 (30.3)	174 (26.6)	0.5212	0.3222	0.7808	0.4966
	CT	19 (50.0)	132 (40.4)	T	53 (69.7)	480 (73.4)				
	TT	17 (44.7)	174 (53.2)							
rs17095830	GG	0 (0.0)	4 (1.2)	G	5 (6.6)	67 (10.2)	0.5854	0.3641	0.4937	0.3138
	AG	5 (13.2)	59 (18.0)	A	71 (93.4)	589 (89.8)				
	AA	33 (86.8)	265 (80.8)							

**Table 7:** Genotype and allele frequencies of rs4552569 and rs17095830 in HLA-B27 (+) AS patients with or without *inflammatory bowel disease* (IBD).

	Genotype	IBD (%)(n = 21)	Without (%)(n = 365)	Allele	IBD (%)(n = 21)	Without (%)(n = 365)	Genotype*P* Value	Dominant*P* Value	Recessive*P* Value	Allelic*P* Value
rs4552569	CC	3 (14.3)	21 (5.8)	C	14 (33.3)	193 (26.7)	0.2986	0.6728	0.1200	0.3494
	CT	8 (38.1)	151 (41.8)	T	28 (66.7)	529 (73.3)				
	TT	10 (47.6)	189 (52.4)							
rs17095830	GG	0 (0.0)	5 (1.4)	G	8 (19.0)	71 (9.8)	0.0443	**0.0250**	0.5877	0.0556
	AG	8 (38.1)	61 (16.8)	A	34 (81.0)	653 (90.2)				
	AA	13 (61.9)	296 (81.8)							

**Table 8:** Difference in the scores of BASDAI, BASFI, and BAS-G among HLA-B27 (+) with AS patients stratified by rs4552569 and rs17095830 genotypes.

SNP	Genotype	BASDAI	BASFI	BAS-G
rs4552569	CC	4.56±1.81	2.13±2.12	4.13±2.52
	CT	4.29±2.17	2.03±2.20	4.52±2.74
	TT	4.25±2.20	2.04±2.21	4.31±2.77
Unadjusted *P*-value		0.7797	0.9762	0.6489
Adjusted *P*-value		0.7790†	0.9082§	0.6448†
rs17095830	GG	3.01±2.64	1.90±4.03	2.94±4.03
	AG	4.61±2.21	2.06±2.02	4.68±2.88
	AA	4.25±2.15	2.06±2.24	4.36±2.70
Unadjusted *P*-value		0.1696	0.9876	0.3200
Adjusted *P*-value		0.1685†	0.9745§	0.3146†
**Two-locus model**				
High-risk group		4.62±2.11^b^	2.03±2.14^b^	4.40±2.83^b^
Low-risk group		4.22±2.17^b^	2.04±2.21^b^	4.38±2.73^b^
Unadjusted *P*-value		0.1738	0.9874	0.9354
Adjusted *P*-value		0.1729†	0.6930§	0.9530†

Data represent means ± S.D..

† Adjusted the effects of age and sex.

§ Adjusted the effects of age, sex and disease duration.

b AS patients with the CC genotype in rs4552569, GG genotype in rs17095830 or heterozygous at both loci were classified into high-risk group and the others were classified into low- risk group.

## Discussion

The first Genome-wide association studies (GWAS) of AS in a European population found an association of two new genetic loci 2p15 (rs10865331) and 21q22 (rs2242944) with AS development, as well as in the SNPs *ANTXR2* (rs4333130) and *IL1R2* (rs2310173), and it confirmed the previously reported associations at *IL23R* (rs11209026) and *ERAP1* (rs27434) [15]. This study revealed that 2p15 (rs10865331), 21q22 (rs2242944), *ANTXR2* (rs4333130) and *IL1R2* (rs2310173) may be susceptibility polymorphisms for AS development, and establishes that IL-23 and IL-1 cytokine pathways may play a major role in AS development [15]. Another GWAS in a European population found that *RUNX3* (rs11249215), *IL12B* (rs6556416), and *LTBR-TNFRSF1A* (rs11616188) are associated with AS development; *ANTXR2* (rs4389526), *PTGER4* (rs10440635), *CARD9* (rs10781500), and *TBKBP1* (rs8070463) are involved in disease pathogenesis, confirming that the above associations. In addition, the *ERAP1* (rs30187) polymorphism which encodes an endoplasmic reticulum aminopeptidase is involved in peptide trimming before HLA class I presentation and only affects ankylosing spondylitis risk in *HLA-B27*-positive individuals [16].

Recently, a GWAS of ankylosing spondylitis in Han Chinese identified two new susceptibility loci, *HAPLN1-EDIL3* (rs4552569) and *ANO6* (rs17095830), both related to bone formation and cartilage development, confirming the above associations with AS [11]. However, *ANTXR2* and *IL23R* are not associated with AS development in Han Chinese [17], [18], [19], [20]. Our results show that rs4552569 and rs17095830 genetic polymorphisms are not associated with AS development and clinical manifestations in the Taiwanese. In the Taiwanese population, the susceptibility genetic polymorphism of the interleukin-12B (*IL-12B*) +1188A/C, which encodes a p40 subunit common to IL-12 and IL-23, may be a risk factor for AS development [20]. Wei et al. [14] also observed that *ORAI1* haplotypes are associated with the development of HLA-B27 positive AS patients. However, these associations have not been validated in the Han Chinese population yet.

The reasons for these inconsistent results in Han Chinese and Taiwanese populations are unknown. Our enrollment criterion for AS patients was a diagnosis by an experienced rheumatologist according to the modified New York criteria (1984), similar to the Han Chinese in the GWAS. Potential healthy controls were randomly selected from patients sequentially admitted to the same medical center for general physical examinations. They resided in the same geographical areas as the AS patients and had no significant medical history or abnormal laboratory results. In the present study, the frequency of rs4552569 C allele and rs17095830 G allele in normal controls (27.9% and 8.7%, respectively) is close to that found in the Han Chinese GWAS controls (28.0% and 10.0%, respectively) [11]. These findings confirm the validity of the genotyping methodology used in this study. Selection bias may have occurred when AS patients with active or inactive disease were enrolled. Because our subjects were adults, these results do not apply to juvenile AS patients. Referral bias was possible, since our data were collected from a single medical center. There is also a concern regarding the relatively smaller sample size in our study. We recognize that small numbers of subjects may limit the statistical power (with 20% statistical power) to detect a small increase in risk. The sample size may result from the limited population in Taiwan. Therefore, larger cohort studies in another population are necessary to provide additional regarding our findings. In summary, rs4552569 and rs17095830 genetic polymorphisms may not be susceptibility factors for AS development and clinical manifestations in the Taiwanese.

## Supporting Information

Figure S1 We examined 12 combinatorial patterns in rs4552569 and rs17095830 gene-gene interaction.
(DOC)
