# Supplementary material for: Association Study of Polymorphisms rs4552569 and rs17095830 and the Risk of Ankylosing Spondylitis in a Taiwanese Population
Source: PLoS One. 2013 Jan 4;8(1):e52801. doi: 10.1371/journal.pone.0052801 (PMC3537770; doi:10.1371/journal.pone.0052801)
Supplement: Figure S1 — We examined 12 combinatorial patterns in rs4552569 and rs17095830 gene-gene interaction. (DOC) [file pone.0052801.s001.doc]

**Supplementary Figure 1**


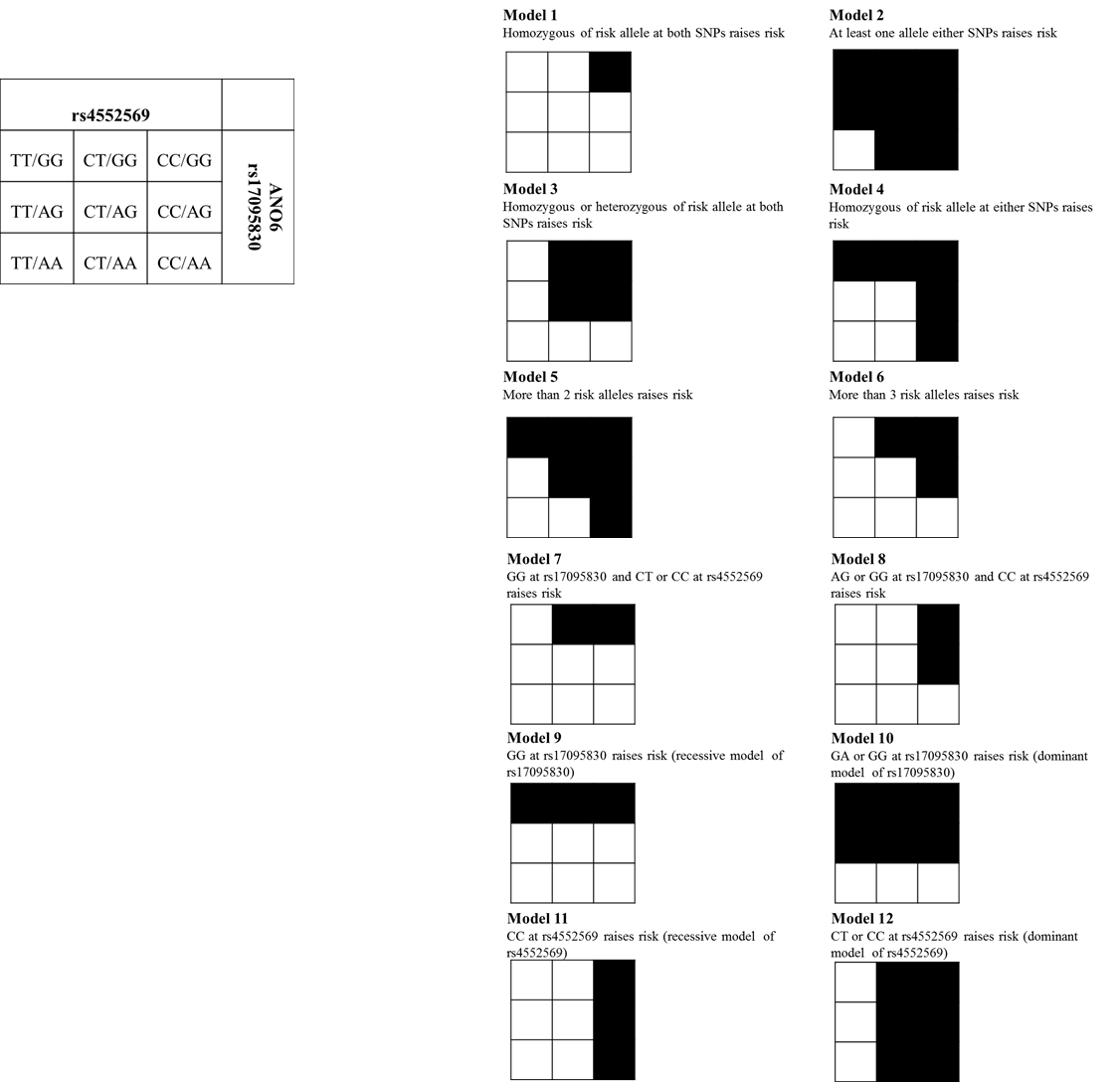


**Supplementary Figure 1**

12 combinatorial patterns in rs4552569 and rs17095830 gene-gene interaction were performed.
